# UCHL3 promotes hepatocellular carcinoma cell migration by de-ubiquitinating and stabilizing Vimentin

**DOI:** 10.3389/fonc.2023.1088475

**Published:** 2023-03-09

**Authors:** Qiancheng Ma, Qiliang Lu, Xiangxiang Lei, Jie Zhao, Wen Sun, Jun Wang, Qing Zhu, Dongsheng Huang

**Affiliations:** ^1^ College of Biotechnology and Bioengineering, Zhejiang University of Technology, Hangzhou, China; ^2^ The Key Laboratory of Tumor Molecular Diagnosis and Individualized Medicine of Zhejiang Province, Zhejiang Provincial People’s Hospital, Affiliated People’s Hospital, Hangzhou Medical College, Hangzhou, China; ^3^ Qingdao Medical College, Qingdao University, Qingdao, China; ^4^ School of Basic Medical Sciences and Forensic Medicine, Hangzhou Medical College, Hangzhou, China; ^5^ The Second Clinical Medical College, Zhejiang Chinese Medical University, Hangzhou, China; ^6^ Department of Emergency and Critical Care Medicine, The First Affiliated Hospital of Xi’an Jiaotong University, Xi’an, China

**Keywords:** HCC, UCHL3, Vimentin, de-ubiquitination, migration, cancer stem cells

## Abstract

**Background:**

Hepatocellular carcinoma (HCC) is a common malignant tumor associated with a poor prognosis. Ubiquitin carboxyl-terminal hydrolase L3 (UCHL3) has been reported to promote diverse tumors, but little is known about its role in HCC.

**Methods:**

Expression levels of UCHL3 in Huh7 and Hep3B cells were measured by qRT-PCR. UCHL3, Vimentin protein levels, and ubiquitination levels were determined by Western blot assay. co-immunoprecipitation, Immunofluorescence, and IHC were used to detect the interaction and expression association between UCHL3 and Vimentin in the cells. Wound healing and Transwell assays were used to measure cell migration. Spheroid formation assay were used to assess stem-like properties.

**Results:**

UCHL3 expression was found to be significantly elevated in HCC and associated with poor prognosis. UCHL3 promoted migration and stem-like properties of HCC cells. Vimentin was identified as a potential de-ubiquitination substrate of UCHL3 and UCHL3 interacted with and promoted the de-ubiquitination of Vimentin, enhancing its stability. Moreover, the suppression of UCHL3 by siRNA or the inhibition by TCID upregulated ubiquitinated Vimentin. Vimentin attenuated the suppression of cell migration caused by knockdown of UCHL3.

**Conclusion:**

UCHL3 was highly expressed in HCC and functioned as an oncogene. Vimentin is a novel substrate of UCHL3 and its stabilization and de-ubiquitination enhanced HCC cell migration.

## Introduction

Liver cancer is the third most common cause of global cancer-related death (1). Over 90% of liver cancer cases are hepatocellular carcinoma (HCC), of which only 5%–15% are candidates for early-stage surgical resection. Chemotherapy and immunotherapy are used to treat advanced HCC, but metastasis leads to unsatisfactory long-term survival rates (2–4). The identification of novel therapeutic targets is thus an urgent matter.

Ubiquitin-like proteins (Ub) consist of 76 amino acids incorporating seven lysine residues, namely, K6, K11, K27, K29, K33, K48, and K63. Mono- or poly-ubiquitinated chains are involved in the regulation of processes such as DNA repair, apoptosis, transcriptional regulation, and endocytosis (5–8). Ubiquitin also play a significant role in HCC pathogenesis, raising the possibility that ubiquitinated components may be therapeutic targets (9, 10). Ubiquitination is dynamic and reversible. De-ubiquitinases (DUBs) remove ubiquitin, protecting their protein substrates from degradation (11, 12). Such enzymes include ubiquitin C-terminal hydrolases (UCHs), including UCHL1/PGP9.5 (protein gene product 9.5), ubiquitin carboxyl-terminal hydrolase L3 (UCHL3), UCHL5/UCH37, and BRCA1-associated protein-1 (BAP1) (13, 14).

Abnormal expression and a dual function of UCHL3 have been described in human cancer. UCHL3 exerts an anti-tumor activity to inhibit the epithelial-mesenchymal transition (EMT) and reduce the stem-cell-like properties of prostate cancer cells (15, 16). However, oncogenic activities have also been reported with UCHL3 being overexpressed in lung cancer (17, 18), ovarian cancer (19), pancreatic cancer (20), and melanoma (21). Aryl hydrocarbon receptor (AhR) (22, 23), FOXM1 (20), lymphoid-specific helicase (LSH) (17), COPS5 (24), and RAD51 (25) are de-ubiquitination targets of UCHL3, which indicate the possibility of UCHL3 being a therapeutic target. However, roles and mechanisms of UCHL3 in hepatocarcinogenesis remain unclear.

Epithelial cells lose polarity and acquire mesenchymal characteristics during the EMT, a process that contributes to tumor cell migration (26). The involvement of Vimentin in the initiation of the EMT has recently been demonstrated (27, 28), and Vimentin is known to be regulated by post-translational modifications, including ubiquitination, phosphorylation, O-linked glycosylation, sumoylation, and ADP-ribosylation (29–31). The ubiquitin ligases, Trim16 (32) and Trim56 (33), have been found to ubiquitinate Vimentin, but less is known about its de-ubiquitination, other than by USP14 (34).

The current study found that UCHL3 was upregulated in HCC patients, indicating a poor prognosis. UCHL3 promoted the migration and stem-like properties of HCC cells. UCHL3 was identified as a DUB, which targets Vimentin, and the consequent de-ubiquitination may be responsible for the promotion of cell migration.

## Materials and methods

### Clinical tissues

Clinical specimens were obtained from patients with hepatocellular carcinoma at Zhejiang Provincial People's Hospital. Freshly excised HCC tissues were fixed using formalin and embedded in paraffin for immunohistochemical (IHC) staining.

### Cell culture

Hep3B, Huh7, and HepG2 cells were obtained from the Cell Bank of the Chinese Academy of Sciences (Shanghai, China) and MIHA from bnbio (Beijing, China). MIHA and Hep3B cells were grown in Minimum Essential Medium (MEM) (VivaCell, C3060-0500) containing 10% fetal bovine serum (FBS) (BI, 04001-1A) and 1% penicillin-streptomycin (Cienry, CR15140). Huh7 and HepG2 cells were grown in Dulbecco’s modified Eagle’s medium (DMEM) (VivaCell, C3060-0500) containing 10% FBS (BI, 04001-1A) and 1% penicillin-streptomycin (Cienry, CR15140). All cells were cultured at 37°C and 5% CO_2_.

### Immunofluorescence colocalization

UCHL3 plasmid (EGFP-UCHL3) (GenePharma, Shanghai, China) were transfected into HCC cells using Lipofectamine 3000 (Thermo Fisher Scientific, L3000-015). Immunofluorescence staining was performed using Vimentin antibody (Proteintech, 10366-1-AP) and Cy3-labeled Goat Anti-rabbit IgG (Beyotime, A0516). Antifade Mounting Medium with 4′,6-diamidino-2-phenylindole (DAPI) (Beyotime, P0131) was used to seal the slices and then photographed under a confocal microscope (Nikon).

### Transfection of plasmids with siRNA/shRNA

Lentivirus-mediated UCHL3 shRNA and NC shRNA (Genomeditech, Shanghai, China) and Vimentin plasmid and siRNAs (100nM) (GenePharma, Shanghai, China) were transfected into HCC cells using Lipofectamine 3000 (Thermo Fisher Scientific, L3000-015).

The target sequences were as follows (5′-3′):

NC: TTCTCCGAACGTGTCACGT;

shUCHL3#1: GGTCAGACTGAGGCACCAAGT;

shUCHL3#2: GGAGGAATCTGTGTCAATGAG;

and for Vimentin (5′–3′):

NC sense: UUCUCCGAACGUGUCACGUTT; NC antisense: ACGUGACACGUUCGGAGAATT;

siVimentin#1 sense: CUGGUUGAUACCCACUCAATT; siVimentin#1 antisense: UUGAGUGGGUAUCAACCAGTT;

siVimentin#2 sense: GCAUCACGAUGACCUUGAATT; siVimentin#2 antisense: UUCAAGGUCAUCGUGAUGCTT.

### Wound healing assay

Cells were cultured in six-well plates until a monolayer formed and a wound created in the middle of the well; cells were washed with 1× phosphate-buffered saline (PBS) to remove debris, and serum-free medium was added. Cell migration was assessed by inspecting the wound area under an inverted microscope (Nikon, DS-Ri2). Images were analyzed by ImageJ software.

### Transwell assay

A total of 600 µl of complete medium was added to the lower chamber of Transwell inserts (Corning), and HCC cells in serum-free medium were inoculated into the upper chamber. Cells were cultured for 24 Hours, and those in the lower chamber were fixed with 4% paraformaldehyde, stained with 0.1% crystal violet, and imaged under an inverted microscope (Nikon, DS-Ri2).

### Spheroid formation assay

Oncospheres were grown in serum-free DMEM-F12 (1:1) medium containing 10 ng/ml epidermal growth factor (EGF), 10 ng/ml fibroblast growth factor (FGF), and N2 (Thermo Fisher, 17502048) for 1–2 weeks and imaged under an inverted microscope (Nikon, DS-Ri2).

### Western blotting and co-immunoprecipitation analysis

Total protein was isolated in Cell lysis buffer for Western and IP (Beyotime, P0013), and concentration was determined by a BCA protein assay kit (Thermo Fisher, 23225). Proteins were separated by PAGE Gel Quick Preparation Kit(10%) (Yeasen, 20325ES62), transferred onto polyvinylidene fluoride (PVDF) membranes (Millipore, IPVH00010), and blocked with 5% skimmed milk (Yeasen, 36120ES76). Primary antibodies raised against UCHL3 (Proteintech, 12384-1-AP), β-actin (Affinity, AF7018), Vimentin (Cell Signal, D21H3), Vimentin (Proteintech, 60330-1-Ig), and Ubiquitin (Santa, P4D1) were added with incubation at 4°C overnight. Membranes were washed three times with TBST (Solarbio, T1082), incubated with horseradish peroxidase (HRP)-conjugated secondary antibody (Beyotime) for 1 h and visualized with ECL reagent (FD). Blots were imaged using the ChemiDoc™ MP Imaging System, and GoldBand 3-color Regular Range Protein Marker (10-180kDa) (Yeasen, 20351ES72) was used to indicate molecular weight.

Cell lysates were prepared in Cell lysis buffer for Western and IP (Beyotime, P0013) for co-immunoprecipitation experiments. Anti-UCHL3 (Proteintech, 12384-1-AP) or -Vimentin antibodies (Proteintech, 10366-1-AP) or IgG (negative control) were added to magnetic beads, according to the manufacturer’s instructions (bimake, B23201).

### Half-life and inhibitor analysis

Cells were treated with 10 μM cycloheximide (CHX; Selleck, S7418) to block protein synthesis, and proteins were extracted at various time points and protein levels assessed by Western blotting for half-life determination.

Hep3B and Huh7 cells were treated with dimethyl sulfoxide (DMSO) or 10 μM TCID (Selleck, S7140) for 24 h, and UCHL3 and Vimentin expression was determined by Western blotting. Western blotting was used to evaluate polyubiquitinated Vimentin following co-immunoprecipitation of Vimentin.

### RT-qPCR

Total RNA was isolated by RNA-Quick Purification Kit (YiShan, RN001) and reverse transcribed into cDNA with PrimeScript™ RT Reagent Kit (Takara, RR037A). SYBR Green PCR Master Mix (Yeasen, 11184ES03) was used for real-time quantitative PCR (RT-qPCR) on a 7500 Real-Time PCR instrument. Relative mRNA expression was normalized by the 2^−ΔΔct^ calculation method. Primer sequences were as follows (5′–3′).

Vimentin:

Forward: AGG CAA AGC AGG AGT CCA CTG A;

Reverse: ATC TGG CGT TCC AGG GAC TCA T;

β-actin:

Forward: TGA CCC AGA TCA TGT TTG AG;

Reverse: CGT ACA GGG ATA GCA CAG;

UCHL3:

Forward: CTGAAGAACGAGCCAGATAC;

Reverse: GCCCATCTACATGAACTAATGC.

### Statistical analysis

Means ± standard deviations for at least three replicates are presented. Statistical analyses were performed by GraphPad Prism 9 (San Diego, CA, USA), and differences were assessed by Student’s t-test. A value of p < 0.05 was considered statistically significant.

## Results

### UCHL3 was upregulated in HCC and associated with poor prognosis

**Figure 1:**
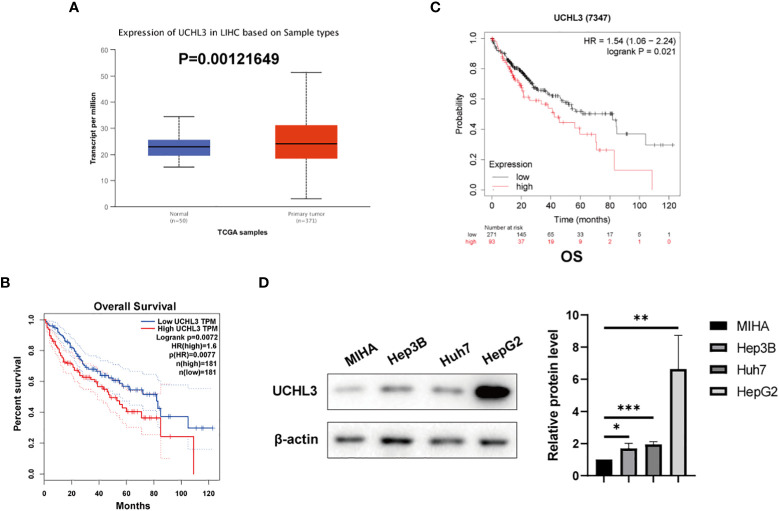
UCHL3 expression and survival analysis in HCC. **(A)** Data relating to primary liver tumors were downloaded from the TCGA database and analyzed by the UALCAN platform (http://ualcan.path.uab.edu/analysis.html) and showed higher UCHL3 expression in tumor tissue. **(B)** Kaplan–Meier analysis by the GEPIA platform (http://gepia.cancer-pku.cn) indicated that HCC patients with increased UCHL3 levels had poorer overall survival. **(C)** Kaplan–Meier analysis (https://kmplot.com/analysis/) of OS showed high UCHL3 expression to be associated with lower patient survival. **(D)** Western blots showed higher UCHL3 expression in HCC cells, Hep3B, Huh7, and HepG2, than in the normal hepatocyte cell line, MIHA. ^*^p < 0.05, ^**^p < 0.01, ^***^p < 0.001.

UCHL3 expression was analyzed by the UALCAN (35) platform and found to be upregulated in HCC by comparison with normal hepatic tissue (**Figure 1A**). Analysis *via* the GEPIA platform indicated that high UCHL3 expression was associated with shorter overall survival among HCC patients (**Figure 1B**). Kaplan–Meier analysis also demonstrated that high UCHL3-expressing HCC patients had reduced survival (**Figure 1C**). UCHL3 was also highly expressed in the HCC cell lines, namely, Hep3B, Huh7, and HepG2, by comparison with the normal hepatocyte cell line, MIHA (**Figure 1D**). In summary, it is suggested that UCHL3 may contribute to liver tumorigenesis and may be a useful prognostic biomarker in HCC.

### UCHL3 overexpression promoted migration and stem-like properties of HCC cells

**Figure 2:**
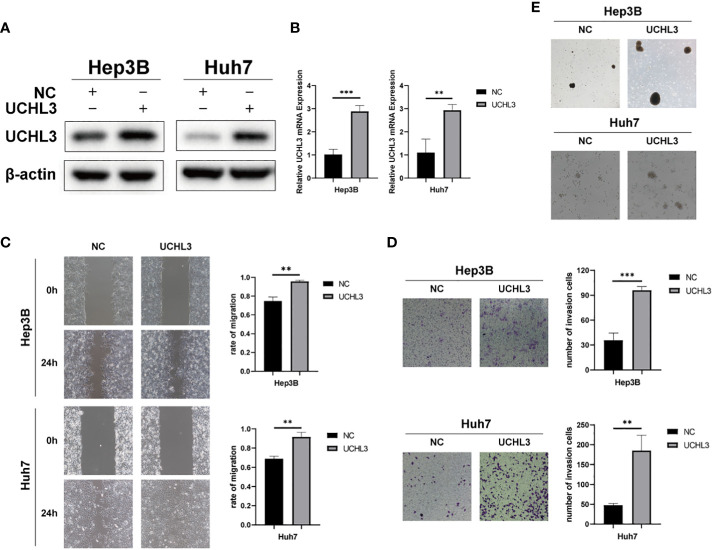
Overexpression of UCHL3 promoted HCC cell migration and stem-like properties. **(A)** UCHL3 overexpression shown by Western blots of Hep3B and Huh7 cells. **(B)** Levels of UCHL3 mRNA in Hep3B and Huh7 cells were confirmed by RT-qPCR. **(C)** UCHL3 overexpression promoted Hep3B and Huh7 migration, as shown by wound healing assay. **(D)** UCHL3 overexpression promoted Hep3B and Huh7 migration, as shown by Transwell assay. **(E)** UCHL3 overexpression promoted spheroid formation in Hep3B and Huh7 cells. ^**^p < 0.01, ^***^p < 0.001.

Huh7 and Hep3B cells stably overexpressing UCHL3 were generated (**Figures 2A, B** **)**. UCHL3 overexpression promoted cell migration during wound healing (**Figure 2C**) and Transwell assays (**Figure 2D**). UCHL3 Overexpressing cells formed larger spheroids, indicating increased stem-like properties, compared with control cells (**Figure 2E**). Overall, UCHL3 may contribute to malignant progression by promoting migration and stem-like properties of HCC cells.

### UCHL3 knockdown inhibited migration and reduced stem-like properties of HCC cells

**Figure 3:**
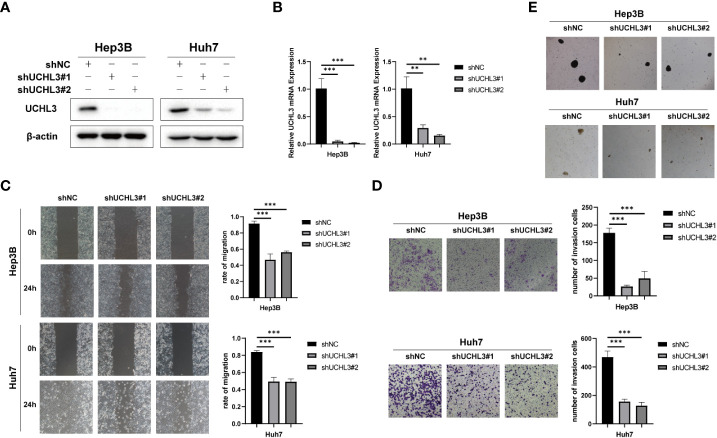
UCHL3 knockdown inhibited migration and stem-like properties of HCC cells. **(A)** Knockdown of UCHL3 in Hep3B and Huh7 cells was confirmed by Western blotting. **(B)** RT-qPCR showed reduced expression of UCHL3 mRNA in Hep3B and Huh7 cells. **(C)** UCHL3 knockdown suppressed Hep3B and Huh7 cell migration during wound healing assays. **(D)** UCHL3 knockdown suppressed Hep3B and Huh7 cell migration during Transwell assays. **(E)** UCHL3 knockdown inhibited spheroid formation in Hep3B and Huh7 cells. ^**^p < 0.01, ^***^p < 0.001.

Stable knockdown of UCHL3 was established in HCC cell lines (**Figures 3A, B** **)**. UCHL3 knockdown inhibited cell migration and stem-like properties, as revealed by wound healing and Transwell assays (**Figures 3C, D** **)** and spheroid formation assays (**Figure 3E**). Loss of UCHL3 thus impaired migration and stem-like properties of HCC cells.

### Identification of Vimentin as a new substrate of UCHL3

**Figure 4:**
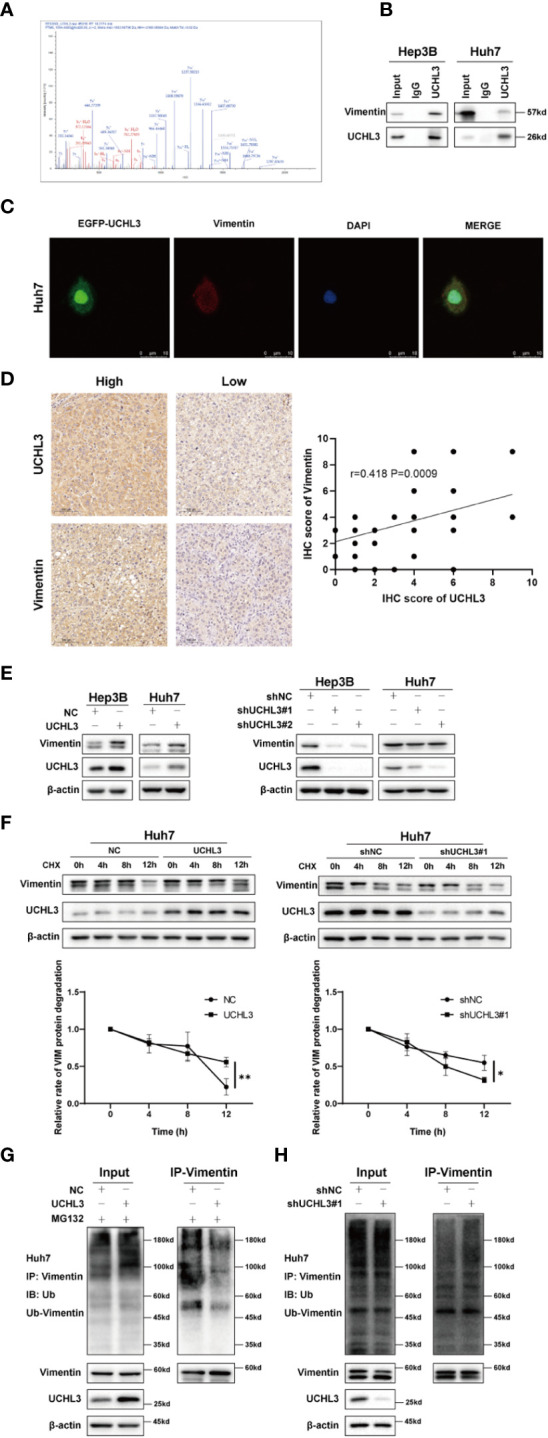
UCHL3 de-ubiquitinylated and stabilized Vimentin. **(A)** Mass spectrometry showed that the UCHL3-binding complex contained Vimentin peptide fragments. **(B)** Co-immunoprecipitation with anti-UCHL3 antibody and Western blotting showed that Vimentin interacted with UCHL3 in Hep3B and Huh7 cells. **(C)** Immunofluorescence showed the colocalization of UCHL3 and Vimentin in cells. **(D)** IHC analysis showed a positive correlation between the expression of UCHL3 and Vimentin in HCC. **(E)** UCHL3 overexpression increased Vimentin levels, and UCHL3 inhibition reduced Vimentin levels, as shown by Western blotting. **(F)** UCHL3 overexpression increased and UCHL3 knockdown decreased the stability of Vimentin protein in Huh7 cells. **(G)** The degree of Vimentin ubiquitination was significantly reduced by UCHL3 overexpression in Huh7 cells, as shown by Western blotting. **(H)** UCHL3 knockdown increased the degree of Vimentin ubiquitination in Huh7 cells, as shown by Western blotting. ^*^p< 0.05, ^**^p < 0.01.

The above results demonstrate the contribution of UCHL3 to migration and stem-like properties of HCC cells, but downstream targets of this de-ubiquitinase remain unknown. Co-immunoprecipitation experiments with UCHL3 antibody followed by mass spectrometry analysis (IP-MS) were performed to identify potential substrates of UCHL3. UCHL3-binding protein complex contained peptide fragments of Vimentin, indicating a Vimentin–UCHL3 interaction (**Figure 4A**). Co-immunoprecipitation experiments in HCC cells confirmed the interaction of UCHL3 with Vimentin (**Figure 4B**). Immunofluorescence results show the colocalization of UCHL3 and Vimentin in cells (**Figure 4C**). The findings suggest that Vimentin is a novel protein-binding partner of UCHL3.

### UCHL3 stabilized Vimentin protein *via* de-ubiquitination

We performed immunohistochemical staining for UCHL3 and Vimentin in HCC tissues. The results showed a positive correlation between the expression of UCHL3 and Vimentin in HCC (**Figure 4D**). Next, cells in which UCHL3 was either overexpressed or knocked down were used to investigate its regulatory impact on Vimentin. UCHL3 promoted Vimentin protein expression, and UCHL3 knockdown inhibited Vimentin protein expression (**Figure 4E**). UCHL3 overexpression enhanced the stability of Vimentin protein, while UCHL3 inhibition suppressed its stability (**Figure 4F**). Consistent with the role of UCHL3 as a DUB, UCHL3 overexpression decreased the ubiquitination level of endogenous Vimentin (**Figure 4G**), and Vimentin ubiquitination was increased when UCHL3 was knocked down (**Figure 4H**). In conclusion, the role of UCHL3 as a DUB allowed stabilization of Vimentin.

### Inhibition of UCHL3 impaired HCC cell migration

**Figure 5:**
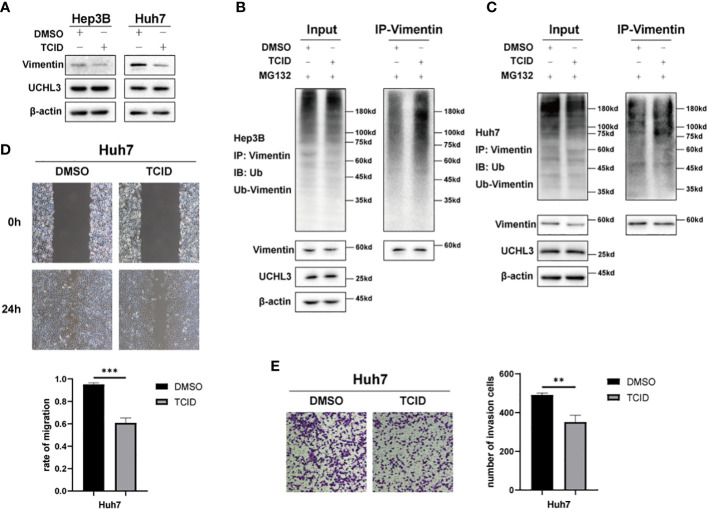
Inhibition of UCHL3 impaired HCC cell migration. **(A)** TCID treatment reduced Vimentin levels in Hep3B and Huh7 cells, as shown by Western blotting. **(B)** TCID treatment promoted Vimentin ubiquitination in Hep3B cells, as shown by Western blotting. **(C)** TCID treatment promoted Vimentin ubiquitination in Huh7 cells. **(D)** TCID inhibited Huh7 migration during wound healing assays. **(E)** TCID inhibited Huh7 migration during Transwell assays. ^**^p < 0.01, ^***^p < 0.001.

TCID is an UCHL3 inhibitor (22) and was found to reduce Vimentin expression without any change in UCHL3 (**Figure 5A**). TCID treatment also significantly increased Vimentin ubiquitination in Hep3B and Huh7 cells (**Figures 5B, C** **)**. In a similar manner to UCHL3 knockdown, TCID treatment also suppressed HCC cell migration (**Figures 5D, E** **)**. In summary, inhibition of UCHL3 by TCID impaired HCC cell migration.

### UCHL3 facilitated HCC cell migration through regulating Vimentin

**Figure 6:**
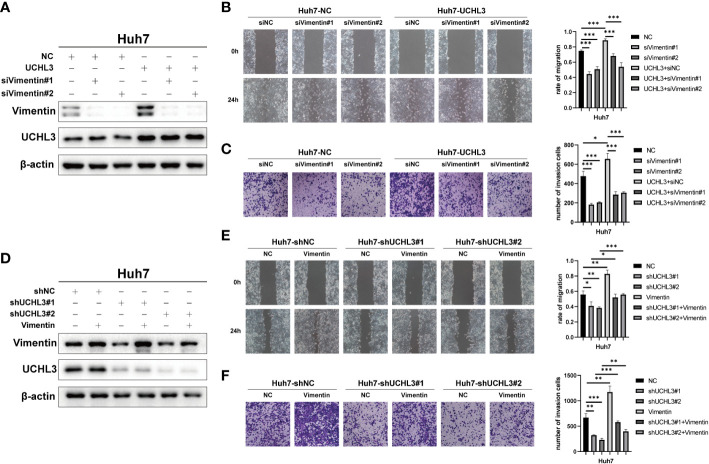
UCHL3 facilitated HCC cell migration through regulating Vimentin. **(A)** Overexpression of UCHL3 and knockdown of Vimentin in Huh7 cells shown by Western blotting. **(B)** Vimentin knockdown abolished the increased cell migration induced by UCHL3 overexpression, as shown by wound healing assays. **(C)** Vimentin knockdown abolished the increased cell migration induced by UCHL3 overexpression, as shown by Transwell assays. **(D)** Knockdown of UCHL3 and overexpression of Vimentin in Huh7 cells shown by Western blotting. **(E)** Vimentin overexpression reversed the inhibition of cell migration caused by UCHL3 knockdown, as shown by wound healing assays. **(F)** Vimentin overexpression reversed the inhibition of cell migration caused by UCHL3 knockdown, as shown by Transwell assays. *p < 0.05, **p < 0.01, ***p < 0.001.

**Figure 7:**
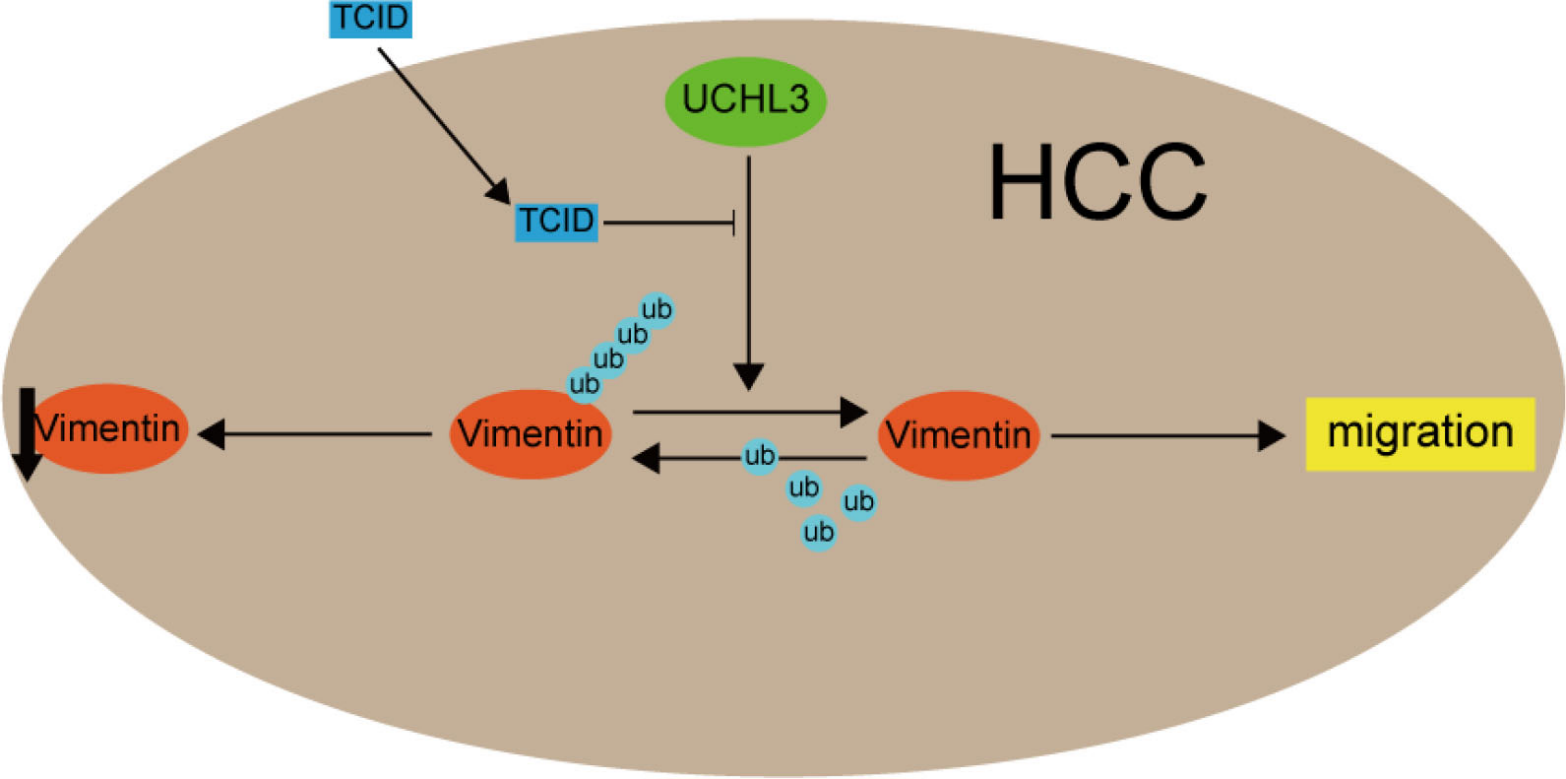
A mechanism model figure of UCHL3 facilitated HCC cell migration through regulating Vimentin.

Functional rescue experiments were performed to demonstrate actions of UCHL3 on Vimentin. Knockdown of Vimentin inhibited HCC cell migration and suppressed UCHL3-induced HCC cell migration, as illustrated by wound healing and Transwell assays (**Figures 6A–C**). Vimentin overexpression promoted HCC cell migration and attenuated the inhibition of cell migration due to UCHL3 knockdown (**Figures 6D–F**). In conclusion, the findings suggest that UCHL3 promotes HCC cell migration through stabilizing Vimentin. Together, the abovementioned results show that the DUB activity of UCHL3 stabilizes the expression of Vimentin and promotes the migration of HCC. And TCID can inhibit the DUB activity of UCHL3 (**Figure 7**).

## Discussion

Worldwide HCC incidence is on the increase and is estimated to rank third for cancer-related deaths by 2030 (36). Indeed, liver cancers, along with pancreatic cancer, generally have low survival rates (37). Ubiquitin dysregulation, due to imbalances between ubiquitinase and de-ubiquitinase activities, is thought to affect tumor development (38–40), but UCH de-ubiquitinases have been rarely studied in HCC, by contrast with USP enzymes (41). Only UCH37 has been investigated in liver cancer cells (42), but other family members may also have an impact. The current study correlated abnormally high UCHL3 expression with the occurrence and malignant development of HCC. UCHL3 promoted HCC cell migration and stem-like properties.

Cell migration and stem-like properties contribute to the aggressiveness of tumors and poor patient prognosis (43). Many proteins involved in HCC cell migration are regulated by ubiquitination, such as β-catenin (44), snail (45, 46), and ZEB1 (47). The EMT marker, Vimentin, may also influence tumor progression. Indeed, the ubiquitination of Vimentin influenced by symmetric dimethylarginine (sDMA) is thought to be necessary for the roles of MTAP and PRMT5 in lung cancer metastasis (48). Salvi et al. identified Vimentin as a molecular chaperone of LASP1 that participates in cancer development (49), and You et al. showed that LASP1 protects Vimentin from ubiquitination and degradation and also interacts with HBX (50). Moreover, BECN1 promoted NSCLC cell migration by regulating USP14-mediated Vimentin ubiquitination (51). AKT signaling, known to be active in cancer cells (52), has been shown to mediate lncRNA VAL binding to Vimentin to eliminate Trim16-dependent Vimentin polyubiquitination and degradation and promote LAD invasion and metastasis (32). Vimentin expression has often been linked to ubiquitination, but the influences of de-ubiquitinases have received less attention. UCHL3 was demonstrated, during the present work, to be a new DUB of Vimentin, which enhanced its stability, an effect that was inhibited by TCID treatment. Functional rescue experiments indicated the dependence of UCHL3-mediated HCC cell migration on Vimentin regulation.

Previous reports have indicated the deubiquitinating and protein-stabilizing effect of UCHL3 in several human cancer types (19, 20, 22) and demonstrated its contribution to carcinogenesis (14). UCHL3 promoted pancreatic cancer cell proliferation and migration by deubiquitinating FOXM1 (20). The Aryl hydrocarbon receptor (AhR) protein was stabilized by UCHL3 de-ubiquitination in NSCLC and promoted stem-cell-like properties (22). In addition, UCHL3 induced tumorigenesis in ovarian cancer by deubiquitinating and stabilizing TRAF2 (19). UCHL3 promoted migration of the liver cancer cells of the current study by deubiquitinating Vimentin. We acknowledge some limitations to the current *in vitro* study, and *in vivo* experiments are needed to confirm the findings.

In conclusion, UCHL3 was found to be highly expressed in HCC and indicated a poor prognosis. The DUB activity of UCHL3 stabilized Vimentin and promoted HCC malignant progression. The present findings give new insights into HCC pathogenesis, indicating that the UCHL3/Vimentin axis may provide an innovative therapeutic target.

## Data availability statement

The original contributions presented in the study are included in the article/supplementary material. Further inquiries can be directed to the corresponding authors.

## Ethics statement

The studies involving human participants were reviewed and approved by Medical Ethics Committee of Zhejiang Provincial People’s Hospital. The patients/participants provided their written informed consent to participate in this study.

## Author contributions

Conception, design, and writing by QM. Investigation, visualization, and methodology by QL, XL, JZ, WS, and JW. Administrative, supervision, or material support by QZ and DH. All authors contributed to the article and approved the submitted version.
